# Healthy Prenatal Diet Associated with Lower Risk of Excessive Gestational Weight Gain in a Predominantly Latina Sample

**DOI:** 10.3390/nu18121953

**Published:** 2026-06-17

**Authors:** Eden Haramati, Catherine Monk, Pamela A. Koch, Daniel Rabinowitz, Randi L. Wolf

**Affiliations:** 1 Program in Nutrition, Department of Health Studies and Applied Education Psychology, Teachers College, Columbia University, 525 West 120th Street, New York, NY 10027, USA; pak14@tc.columbia.edu (P.A.K.); wolf@tc.columbia.edu (R.L.W.); 2 Departments of Obstetrics & Gynecology and Psychiatry, Columbia University, 622 West 168th St. PH15, New York, NY 10032, USA; cem31@cumc.columbia.edu; 3 Department of Statistics, Columbia University, 1255 Amsterdam Avenue, New York, NY 10027, USA; dr105@columbia.edu

**Keywords:** prenatal diet quality, healthy eating index, gestational weight gain, Latina pregnant women

## Abstract

**Background/Objectives**: More than half of all pregnant women in the United States exceed gestational weight gain (GWG) recommendations, which is associated with negative maternal and child health outcomes. The aim of this study was to examine the relationship between prenatal diet quality, measured with the Healthy Eating Index (HEI)-2020, and odds of excessive GWG among a predominantly Latina sample living in the US. **Methods**: This was a secondary data analysis from a longitudinal study and included 118 pregnant women between the ages of 18 and 45. Logistic regression models were used to investigate if higher scores of the HEI components were associated with a reduced risk for excessive GWG. **Results**: About 17% of the sample was classified with inadequate GWG, 22% with adequate GWG, and 61% with excessive GWG. The mean total HEI score for the total sample was 54.1 out of 100. (Higher total HEI scores suggest better dietary quality.) When compared with the scores of the inadequate and excessive GWG groups, the adequate GWG group had significantly higher total HEI scores (58.4) (*p* < 0.05), as well as specific HEI component scores: Greens and Beans (3.5) and Seafood and Plant Proteins (3.5) (*p* < 0.01). A higher score on these HEI components was associated with a lower risk of excessive GWG. **Conclusions**: Meeting the recommended daily servings of greens and beans, as well as seafood and plant proteins, may help reduce the risk of excessive GWG.

## 1. Introduction

In 2009, the Institute of Medicine (IOM) and the National Research Council (NRC) updated the Gestational Weight Gain (GWG) guidelines [1]. The GWG guidelines provide recommendations on ranges of adequate weight gain during pregnancy, which vary according to a woman’s pre-pregnancy body mass index (pBMI). Gaining weight within these ranges is considered adequate; above these ranges is considered excessive; and below these ranges is considered inadequate GWG. In the United States, excessive GWG is quite common, with almost half of women falling within this category [2] and is emerging as a “worldwide epidemic,” with a global prevalence of about 45.5% [3]. Excessive GWG is associated with various negative health outcomes for both mother and infant, such as pre-eclampsia, cesarian delivery, postpartum weight retention, macrosomia, large-for-gestational age, and higher risk for childhood obesity, among many others [4]. Currently, public health efforts target reducing excessive GWG to achieve adequate (ideal) GWG, as excessive GWG is more common than inadequate GWG [5,6].

Given the adverse consequences of excessive GWG and its high prevalence within the US population, to promote optimal health outcomes for mothers and their children, it is imperative to identify the modifiable determinants of GWG and provide prevention strategies for excessive GWG [3].

The Institute of Medicine and the National Research Council categorize the identified determinants of GWG into three categories: (1) social/built/natural and life-stage environment (e.g., access to healthy food, opportunities for physical activity, partner and family support); (2) maternal factors (e.g., sociodemographic factors, anthropometric factors, medical history); and (3) energy balance/nutrients (e.g., food, nutrient, and energy intake). One of the modifiable determinants of GWG is prenatal dietary intake. The relationship between prenatal diet quality and the adequacy of GWG has been recently studied. Prenatal diet quality has been measured in research with various indices, such as the Healthy Eating Index (HEI) [7,8,9,10,11,12], modified versions of the HEI [6,13,14], or other indices [10,15,16,17,18,19,20], including ones that were designed specifically for a particular region, taking into consideration the local food recommendations. These indices use different scoring systems to measure the overall nutritional quality of food intake. Despite the utilization of different indices to measure prenatal diet quality, many studies suggest that high diet quality may be protective against excessive gestational weight gain [7,10,11,12,13,15,16,17,18,20]. As such, prenatal diet quality demonstrates importance as a determinant of GWG.

However, research suggests that disparities in diet quality exist between women of different ethnicities [21,22]. Among a US cohort of women during the periconception period, Hispanic women had a mean total HEI score of 61 out of 100, non-Hispanic Black women had a mean score of 54, whereas non-Hispanic White women had a mean score of 65 [21]. Another study [22] found that among a US cohort, Hispanic women’s mean total HEI scores during the periconception–first trimester was 66 (an overall diet that is considered suboptimal [23]), which was similar to the mean total HEI score for non-Hispanic White women (67) and Asian Pacific Islander women (67) but higher than the mean total HEI score of non-Hispanic Black women, who had a score of 59 (an overall diet that is considered poor [23]). During the second and third trimesters, all total HEI scores dropped across the subgroups, but the inequities remained mostly similar [22]. Findings suggest that women of color may be at higher risk of poor prenatal diet quality and thus may be at higher risk of suboptimal gestational weight gain.

However, to date, no studies explore prenatal diet quality and its association with gestational weight gain among Latina women within the United States. To address this critical gap in the literature, this study examines the associations of diet quality with the adequacy of GWG among a sample of pregnant women who were predominantly Latina (67%) and living within the U.S.

## 2. Materials and Methods

### 2.1. Study Design

This study is a secondary data analysis of data collected from 2011 to 2017 in the Prenatal Stress: The Epigenetic Basis of Maternal and Perinatal Effects study conducted at the Perinatal Pathways research lab at Columbia University Irving Medical Center (CUIMC) in New York City, NY, USA. The overall aims of this parent study were to determine whether pregnant women’s distress affects epigenetic gene regulation related to perinatal development, identify the perinatal consequences of pregnant women’s distress, and ascertain the causal influence of epigenetic modification on the neurodevelopment of offspring [24].

Participants were recruited from the Departments of Obstetrics and Gynecology at CUIMC. Inclusion criteria included enrollment in standard prenatal care, as well as self-reported good health of mother and fetus. Exclusion criteria included smoking, use of recreational drugs, lack of fluency in English, pregnancy with multiples, or reported use of nitrates, steroids, beta blockers, triptans, or psychiatric medications.

A total of 187 participants were enrolled in the original study. Ethnicity was self-identified. Participants were asked “what ethnicity do you consider yourself?” and selected from the following options: Hispanic/Latina or not Hispanic/Latina. The original sample was a group of predominantly Latina (69%) pregnant women between the ages of 18 and 45. Participants were followed longitudinally over pregnancy and postpartum. At three points during pregnancy, weight and dietary intake data were collected. (The targeted weeks for research visits were 12–22 gestational weeks, 24–27 gestational weeks, and 34–37 gestational weeks.) Height was self-reported.

Dietary intake data was obtained via an internet-based automated self-administered 24 h dietary recall (ASA24, a record of all foods and beverages consumed in the previous day). The ASA24s were completed by participants at the Perinatal Pathways lab.

**Figure 1:**
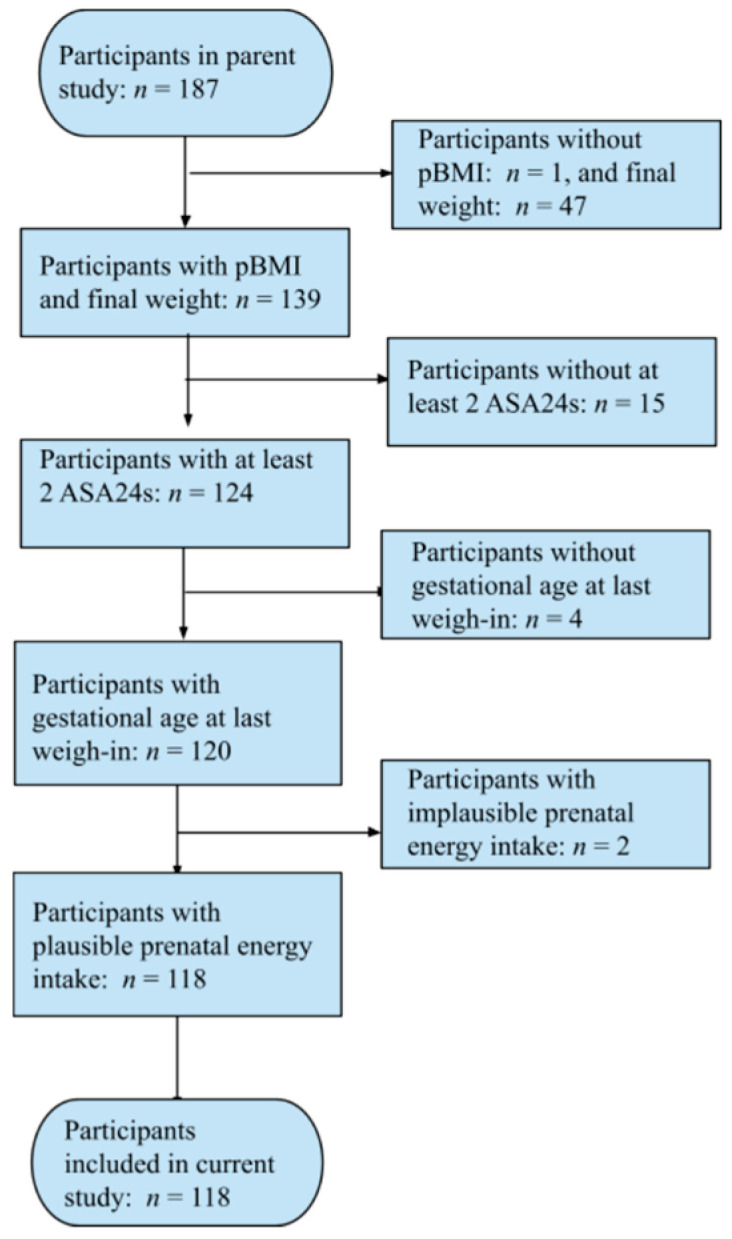
Flowchart of sample screening for current study.

The following criteria were used to select participants ineligible for the current study: participants who had missing data (on pBMI, maternal weight at 34–37 weeks, or the specific gestational age when the last maternal weight was measured); or reported caloric intake outside of a plausible range < 2510.4 kJ (<600 kcal) or >20,920 kJ (>5000 kcal); or did not have at least two ASA24s collected (see Figure 1). The final number of participants eligible for analysis in the current study was n = 118. In the current study, acculturation was operationalized with the primary language spoken by the participant. A response in English represented “more acculturated,” whereas any other response option was considered “less acculturated.”

### 2.2. Variables

#### 2.2.1. Diet Quality: HEI-2020 Total and Component Scores

Data regarding the foods and beverages consumed by participants were obtained using the 2011 version of the ASA24 (ASA24-2011, Rockville, MD, USA). Multiple ASA24s were collected for each participant [25]. The ASA24s were used to calculate HEI-2020 scores to assess diet quality. The total HEI score measures adherence to the 2020–2025 version of the Dietary Guidelines for Americans on a total scale of 0–100 [26]. There are 13 components of the HEI, divided into “adequacy” and “moderation” components. Individual HEI components are measured on a scale of 0–5 or 0–10, which varies based on the component. The maximum score for each of these subcategories is listed alongside each component.

The adequacy components include Total Fruits (5); Whole Fruits (5); Total Vegetables (5); Greens and Beans (5); Whole Grains (10); Dairy (10); Total Protein Foods (5); Seafood and Plant Proteins (5) and Fatty Acids (10). The moderation components include Refined Grains (10); Sodium (10); Added Sugars (10) and Saturated Fats (10).

The highest score (5 or 10, depending on the component) is achieved for adequacy components by meeting or exceeding the recommendation. Any score between zero and the maximum score for each component reflects the level of adherence with the recommendation, with higher scores reflecting higher intakes. The highest score (5 or 10, depending on the component) is achieved for moderation components by not exceeding the recommendation or consuming less than the limit. Any score between zero and the maximum score for each component reflects the level of adherence with the recommendation, with higher scores reflecting lower intakes. Higher scores on components and higher total scores (which is a sum of the scores on all of the components) reflect better adherence to the Dietary Guidelines for Americans.

Total HEI-2020 scores and scores for the components of the HEI were calculated with SAS version 9.4 (Cary, NC, USA) using the *HEI ASA24* SAS script provided by the National Cancer Institute (NCI) on the NCI website [27].

HEI scores were computed for each ASA24 completed. Fifty-five participants had 2 ASA24s collected, and 63 participants had 3 ASA24s collected. The total distribution of recalls was: Time Point 1 (12–22 gestational weeks): 71 recalls; Time Point 2 (24–27 gestational weeks): 113 recalls; and Time Point 3 (34–37 gestational weeks): 115 recalls.

A mean HEI score was generated for each participant by averaging the (two or three) total HEI scores from each participant to create a variable reflective of diet quality during pregnancy, as has been done in other studies [9,17].

#### 2.2.2. Adequacy of GWG

Adequacy of GWG categories (inadequate, adequate or excessive) was defined according to IOM recommendations. The IOM guidelines indicate specific ranges for adequate GWG based on pre-pregnancy BMI: 12.70–18.14 kg with an underweight BMI (<18.5 kg/m^2^), 11.34–15.88 kg for a normal BMI (18.5–24.9 kg/m^2^), 6.80–11.34 kg for an overweight BMI (25.0–29.9 kg/m^2^) and 4.99–9.07 kg for an obese BMI (≥30.0 kg/m^2^).

The IOM provides guidelines for total GWG and rates of GWG (per week) for the second and third trimesters based on pBMI.

Information about total GWG at the point of delivery was not available for the sample in the current study. The protocol for this research study indicated that weight would be measured at 34–37 weeks. (However, final weights were collected between 31 and 39 gestational weeks.) Therefore, a gestational weight was measured at some point during the third trimester for all of the participants.

To our knowledge, there is not a standardized method for categorizing the adequacy of gestational weight gain in the literature for similar research studies. Therefore, to determine the adequacy of GWG (i.e., inadequate, adequate or excessive) a set of formulas was constructed to generate a lower bound and upper bound GWG range for each pBMI category. These formulas factor in the time point (number of gestational weeks) that the weight was taken. These formulas were constructed after consultation with experts in the field of human physiology and obstetrics.

To determine an adequate GWG range, the calculation is done on a patient-by-patient basis. A first-trimester weight gain of 2 lb was suggested to be used. The upper bound and lower bound rates of weight gain are used based on the pBMI of the individual patient.

However, using a 2 lb first-trimester GWG assumption does not generate the same total adequate GWG ranges that are suggested by the IOM for each pBMI. To address this issue, the first-trimester GWG assumption for the lower bound and upper bound GWG for each pBMI can be modified to generate adequate GWG ranges that are consistent with the suggested IOM GWG. GA refers to the number of gestational weeks when participants’ final weights were measured for the parent study.

Underweight
Lower bound: (1) + (GA − 13) × (1)
Upper bound: (4.9) + (GA − 13) × (1.3)
Normal
Lower bound: (3.4) + (GA − 13) × (0.8)
Upper bound: (8) + (GA − 13) × (1)
Overweight
Lower bound: (1.5) + (GA − 13) × (0.5)
Upper bound: (6.1) + (GA − 13) × (0.7)
Obese
Lower bound: (0.2) + (GA − 13) × (0.4)
Upper bound: (3.8) + (GA − 13) × (0.6)

The total GWG for each participant was computed as the difference between each participant’s self-reported pre-pregnancy weight and the last measured weight (during the third trimester). The total weight gain was categorized as inadequate if it fell below the adequate range; adequate if it fell within the adequate range; or excessive if it exceeded the adequate range.

#### 2.2.3. Statistical Analysis

STATA 18.0 Basic Edition (StataCorp LLC, College Station, TX, USA) statistical software was used to analyze the data. Summary statistics and simple tests of association were used to describe the demographic characteristics of the study sample. For continuous characteristics: sample means and sample standard deviations (SDs) in the sample and by GWG adequacy groups (inadequate GWG, adequate GWG, and excessive GWG) were reported. Frequencies were reported for discrete characteristics and for discretized versions of some continuous characteristics for the total sample and for each GWG adequacy group. Univariate one-way Analysis of Variance (ANOVA) analyses were used to compare means across GWG adequacy groups. Chi-square tests of association were used to examine associations between discrete measures and GWG adequacy groups.

Means and standard deviations were reported for the mean total HEI scores and the average HEI component scores, for both the total sample and also by GWG group (inadequate GWG: n = 20; adequate GWG: n = 26; and excessive GWG: n = 72). To investigate differences in mean total HEI scores and mean HEI component scores across the GWG groups, a one-way ANOVA was used. The Kruskal–Wallis tests were used to verify the results of the ANOVA.

In the following analyses, the adequate GWG group (n = 26) was used as the reference group for the excessive GWG group (n = 72). Due to the fact that the focus of these analyses was on factors associated with excessive GWG, as compared to adequate (ideal) GWG, the inadequate GWG group (n = 20) was excluded from these analyses. Logistic regression models were used to investigate if higher overall diet quality based on the total HEI was associated with a reduced risk for excessive GWG. Inclusion of covariates and interaction terms in the final model was contingent upon statistical significance of a candidate regressor in simple regression models (*p* < 0.05), the effect that the inclusion of a variable would have on inferences regarding the predictor of interest, or the plausibility of the estimated regression parameters. In these analyses, the reference group for the income variable was $0–$25,000 and the reference group for the education variable was High School/GED (General Educational Development Test), which were the lowest categories for these variables. The reference group for the ethnicity variable was “not Hispanic/Latina,” and the reference group for the race variable was “White/Caucasian” to align with the primary research questions.

Logistic regression models were used to investigate if higher scores of the HEI components were associated with a reduced risk for excessive GWG. Multiple regression analyses examined the association of each of the HEI components with the outcome. Each model was adjusted for pBMI, maternal age, education, income, race, and ethnicity.

## 3. Results

### 3.1. Descriptive Statistics

Table 1 displays the characteristics of the total sample and by gestational weight gain (GWG) group (inadequate, adequate, and excessive GWG). The means and standard deviations are reported for continuous variables, and percentages and frequencies are reported for discrete variables.

Across the total sample, 17% of participants had inadequate GWG (n = 20); 22% had adequate GWG (n = 26); and 61% had excessive GWG (n = 72).

Average maternal age was 29.9 (SD = 6.1) years. The mean pBMI was 25.8 (SD = 5.3), indicative of an average pBMI in the overweight range. Approximately 41% of participants had no children, 52% of participants had 1–2 children, and 7% had three or more children. Education level varied across the sample. The percentage in the lowest household income category was slightly higher than the percentages in the higher income categories.

Approximately 68% of the sample were classified as more acculturated (primary language spoken was English versus any other language) versus less acculturated (primary language spoken was a language other than English).

### 3.2. Diet Quality and Odds of Excessive Gestational Weight Gain

Table 2 presents information about the average total HEI scores and ranges within the total sample and by GWG group. The mean total HEI score was 54.1 (with scores potentially between 0 and 100). The average total HEI scores for the total sample ranged from 28.8 to 85.3.

Table 3 includes the results of the final model. Several variables were selected for inclusion in the final model due to univariate associations with excessive GWG (pBMI, education, income, and ethnicity, *p* < 0.05); importance based on the literature (maternal age); inferences that could be made from the results with its inclusion; or an alternative analysis that supported its inclusion in the model (race).

After adjustment for the selected confounders, the average total HEI score was not statistically significantly associated with excessive gestational weight gain. The test of whether the association between total HEI and risk of excessive gestational weight gain is different with variation in pBMI found no statistically significant evidence of different associations (*p* = 0.14), and therefore an interaction of pBMI and total HEI was not included in this final model.

### 3.3. Diet Quality Components and Odds of Excessive Gestational Weight Gain

The average HEI component scores and ranges are presented in Table 4 for the total sample (n = 118) and by GWG group.

There is a maximum score of 5 for the following HEI components: Total Fruits, Whole Fruits, Total Vegetables, Greens and Beans, Total Protein Foods and Seafood and Plant Proteins. There is a maximum score of 10 for the following HEI components: Whole Grains, Dairy, Fatty Acids, Refined Grains, Sodium, Added Sugars and Saturated Fats.

The average total HEI score, Greens and Beans score, and Seafood and Plant Proteins score were statistically significantly different across GWG groups.

The adequate GWG group had the highest mean total HEI score of 58.4 (SD = 10.8), compared with the excessive GWG group score of 52.3 (SD = 11.2) and the inadequate GWG group score of 55.0 (SD = 7.2).

The adequate GWG group had the highest mean Greens and Beans score of 3.5 (SD = 1.4), compared with the excessive GWG group score of 2.1 (SD = 1.8) and the inadequate GWG group score of 2.9 (SD = 1.7).

Additionally, the adequate GWG group had the highest mean score of Seafood and Plant Proteins, 3.5 (SD = 1.5), compared with an excessive GWG group score of 2.4 (SD = 1.6) and an inadequate GWG group score of 2.6 (SD = 1.4).

Table 5 presents the multiple logistic regression of the HEI components on risk of excessive GWG, with each model adjusted for pre-pregnancy BMI, maternal age, ethnicity, race, education and income. The reference group for this analysis is the adequate GWG group (n = 26) for comparison with the excessive GWG group (n = 72). The logistic regression of excessive GWG versus adequate GWG on HEI components Greens and Beans and Seafood and Plant Proteins indicated strong estimated negative associations with excessive GWG, OR = 0.61, ${\;\chi }_{1}^{2}$ = 8.07, *p* < 0.01 and OR = 0.60, ${\;\chi }_{1}^{2}$ = 7.84, *p* < 0.01, respectively. For every 1-point increase in the Greens and Beans score, there was a 39% lower odds of excessive GWG. For every 1-point increase in the Seafood and Plant Proteins scores, there was a 40% lower odds of excessive GWG.

## 4. Discussion

In the total sample, there was low levels of healthy eating and excessive GWG. The average total HEI score across the sample was 54.1 (SD = 10.7, range = 28.8–85.3). According to the HEI grading system, this total HEI score would be classified as an “F” [28], which is the lowest score bracket (0–59) and indicative of great need for improvement.

Based on NHANES data from 2013 to 2018, the average total HEI score among pregnant women in the US is 63 [29]. Additionally, NHANES data indicates that the total HEI score for all Hispanics (ages 2 and older) was 60 [30]. Other studies in the US found that among Hispanic women during the periconception/prenatal period, total HEI scores ranged from 53 to 62 [21,22,31]. This suggests that the diet quality of the sample in this study was relatively low when compared with pregnant women in the US, the general Hispanic population in the US, and Hispanic women during the periconception/prenatal period.

The prevalence of excessive GWG was also high in this sample: 61% (n = 72) had excessive GWG. Data from a nationally representative sample of pregnant women (based on NHANES data 2003–2016) indicated that, across the three trimesters, 19.6% had inadequate GWG, 30.6% had adequate GWG and 49.8% had excessive GWG [6]. Research with Latina women in the US estimates the prevalence of excessive GWG to be 45–54% [32,33,34]. It appears that the prevalence of excessive GWG in this current study was higher than the prevalence of excessive GWG in both the general US population and in studies with Latina women in the US.

In this study, after adjustment (for pBMI, maternal age, race, ethnicity, education and income), a higher score on the HEI Greens and Beans component or a higher score on the HEI Seafood and Plant Proteins component was associated with lower odds of excessive GWG.

Although the average total HEI score was not associated with the odds of excessive GWG after adjustment for covariates, these two components remained significantly associated with odds of excessive GWG after adjustment for confounders. The total HEI score includes several components that, while important for general good health, may not have any substantial influence on gestational weight gain, such as sodium intake, intake of dairy, ratio of unsaturated fatty acids to saturated fatty acids, etc. As such, it is not surprising that intake of specific components of the HEI, Greens and Beans intake and Seafood and Plant Proteins, were associated with odds of excessive gestational weight gain. While the total HEI score offers valuable information that can be used to rank overall dietary quality, analysis of each of the HEI components is important. A validation study of the HEI-2015 indicated that the individual components offer independent information about dietary intake, which are not all highly correlated with one another or with the total HEI score [35]. Components of the HEI are key to understanding GWG and advising pregnant women on healthy eating relevant to weight gain.

Greens and beans are plant foods that are rich in micronutrients, good sources of fiber, and not energy-dense. Therefore, this category of foods can improve one’s nutrition profile without a large number of calories and may help prevent excessive weight gain by inducing satiety.

The Seafood and Plant Proteins category is a similarly important building block of a balanced dietary pattern for its contribution of an essential macronutrient, protein. Plant proteins also provide added fiber, and seafood proteins offer a variety of micronutrients and omega-3 fatty acids, which make them both excellent protein options. Sufficient intake of protein promotes satiation and can therefore also reduce the risk of excessive GWG.

Other studies have shown that total HEI scores were unrelated to GWG, but specific components of HEI were associated with GWG. A study that evaluated prenatal dietary intake with the HEI-2005 found no significant difference in total HEI-2005 scores across GWG groups but found that insufficient consumption of total vegetables, <1.1 *cup* eq/4184 kJ (1000 kcal), was associated with excessive GWG (*p* = 0.03) [7]. Additionally, another study did not find an association between total HEI-2015 scores and GWG. However, they discovered that greens and beans were negatively associated with GWG in the third trimester (β (SE) = 1.67 (0.76), *p* = 0.04) [11]. Lastly, another study found that participants with excessive GWG had the lowest Greens and Beans and Total Vegetable scores in early pregnancy [12].

The excessive GWG group had a score of 2.1 points (out of a maximum of 5) on the Greens and Beans component. An increase of 0.08-*cup* equivalent of greens and beans would raise this HEI component score by 1 point. Additionally, the excessive GWG group had a score of 2.4 points (out of a maximum of 5) on the Seafood and Plant Proteins component. An increase of 0.32 *oz* equivalent of seafood and plant proteins daily would contribute a 1-point increase in this component. (These calculations were based on the average 8301 kJ/1984 kcal per day consumed by the excessive GWG group during pregnancy.) These dietary modifications can be achievable goals for pregnant women and may offer protection against excess GWG, underscoring the clinical significance of these findings.

Although all of the components of the HEI are important for promoting good health, the findings from this study and the aforementioned studies [7,11,12] suggest that emphasizing intake of specific food groups during pregnancy (i.e., Greens and Beans, Seafood and Plant Proteins, and Total Vegetables) may be higher priorities when aiming to reduce odds of excessive GWG.

ASA24 data from the sample revealed specific foods that contributed to higher intake of greens and beans, as well as seafood and plant proteins. Related to the Greens and Beans component, different variations of rice with beans were a popular choice. Some of the different types of rice with beans included rice with beans and vegetable oil; black, brown, or bayo beans; dry, cooked, or fat added in cooking; rice with beans and beef; rice with beans without fat; and rice with beans and pork with vegetable oil. It is important to note that the rice and beans dish is rich in nutrients but also has the potential to be high in sodium and saturated fat. Interventions can encourage consumption of this nutrient-dense dish but educate participants on alternative methods of preparation that use a lower quantity of fat; unsaturated fats in place of saturated fat; reduced sodium; and all-natural seasonings.

Additionally, leafy green vegetables were commonly consumed in this sample. These included raw lettuce, spinach, endive, chicory, escarole, romaine lettuce, and mixed salad greens. Consumption of these items should be encouraged while also discussing additional items that may be added to enhance flavor (i.e., dressings that are lower in saturated fat, sodium and sugar).

Pertaining to the Seafood and Plant Proteins component, some items consumed included nut mixtures with dried fruits and seeds and peanut butter. A popular fish item was salmon (baked or broiled with vegetable oil). These nutrient-dense options should be encouraged, and participants may also enjoy being exposed to other types of nutritious foods that would enhance their intake of seafood and plant proteins, such as other types of fish and nut butters.

Interventions should include culturally relevant information and recipes/cooking demos that incorporate these specific dietary categories.

There were several limitations in this study. This study may have limited generalizability. Firstly, one of the exclusion criteria for participating in the parent study was lacking fluency in English. Therefore, the results may not be generalizable to Latina women in the US who lack fluent speech in English. Also, one of the exclusion criteria for the current study was missing data on maternal weight at 34–37 weeks (T3). This led to an exclusion of 47 participants, whose last recorded weight was taken before T3 (31–39 gestational weeks). As a result, the sample in the current study may have been restricted to those who were closer to a full-term pregnancy, and therefore the sample may have been skewed to include participants with healthier pregnancies. Additionally, pre-pregnancy weight was based on self-report, and therefore the pre-pregnancy BMI categorization for all participants was subject to misclassification. However, research suggests that there is a strong correlation between self-reported and measured pre-pregnancy weight [36]. Furthermore, although weight immediately before delivery is ideal for calculating total gestational weight gain, this data was not accessible for this study. Therefore, the last measured weight for the parent study was used to calculate gestational weight gain, which has been done in other studies as well [8,9,10,12,18,19]. Lastly, statistical power and representativeness may have been weakened due to the exclusion of participants from the original sample who had missing data or implausible food intake.

There were several strengths of this study. Firstly, repeated measures of dietary intake were obtained during pregnancy at the beginning of the second, end of the second, and beginning of the third trimesters. Therefore, each participant included in this secondary data analysis had two to three dietary recalls. As there is within-person variability in food intake, one recall is typically not representative of overall dietary intake, and repeated measures are necessary to capture information about one’s eating habits. Secondly, the ASA24-2011 was used to collect dietary intake, which is a tool that has been widely used among Hispanic samples to collect dietary data [37,38]. Thirdly, weight during pregnancy was measured on a standardized scale by study staff. Lastly, this study is composed of predominantly Latina adult pregnant women in the US, which addresses an important gap in the literature on prenatal dietary intake and GWG.

## 5. Conclusions

Improving overall diet quality through adherence to the Dietary Guidelines for Americans is an important goal in prenatal care to meet nutritional recommendations during pregnancy, lower the risk of chronic diseases and improve health outcomes for mothers and their children. As the global food environment increasingly reflects the American-style landscape, characterized by an abundance of highly processed foods with little nutritional value, other countries face a similar risk of declining diet quality. Consequently, pregnant women worldwide may become more vulnerable to the same poor dietary patterns and adverse health consequences impacted by excessive gestational weight gain.

Interventions for pregnant women have been successful in improving prenatal diet quality [39]. Therefore, interventions should be designed to be culturally inclusive and relevant. Interventions for Latina pregnant women should emphasize greens and beans and seafood and plant protein intake in counseling and education (such as through recipes/cooking demos) to reduce the risk for excessive GWG. Intake of approximately a ½ *cup* equivalent of greens and beans daily, such as through dark green vegetables, beans, peas, and lentils [40], could lead to the attainment of the maximum score for this component. Additionally, consuming about 1.5 *oz* of seafood and plant proteins daily, such as through seafood, nuts, seeds, soy products (other than beverages), and beans, peas, and lentils [40], could lead to the maximum score for this component. (All of these recommendations assume a caloric intake of about 8368 kJ/2000 kcal daily but should be adjusted based on the energy intake of the individual.) However, even smaller improvements in these components can be beneficial and lower the risk of excessive GWG.

Furthermore, in the United States, healthcare providers should encourage participation in the Special Supplemental Nutrition Program for Women, Infants, and Children (WIC) for women who are eligible to improve access to nutrient-dense foods and nutrition education. Participation in the WIC program and dietary interventions can help address disparities in prenatal diet quality through developing food-related knowledge and skills and facilitating access to nutritious foods. The increased diet quality may thereby improve the likelihood of optimal maternal and child health outcomes for Latina women.

The results of this study relate to Latina pregnant women living in the US. The extrapolation of the findings is limited due to the exclusion criteria for the parent study, which excluded individuals who lacked fluency in English.

While these results do not indicate causality between dietary intake and gestational weight gain and may not be generalizable to all pregnant Latina women, the findings are suggestive of associations between dietary intake of specific foods and excessive gestational weight gain. Nevertheless, the benefits of improved dietary quality during pregnancy are well-established and should be supported worldwide.

## Figures and Tables

**Table 1:** Sample characteristics.

Gestational Weight Gain Groups									
	Total Sample		Inadequate		Adequate		Excessive		
	100% n = 118		17% n = 20		22% n = 26		61% n = 72		
Characteristics	M	SD	M	SD	M	SD	M	SD	Test Statistic (*p*-Value)
Maternal age, years	29.9	6.1	30.2	5.0	31.5	6.5	29.2	6.2	F = 1.34 (0.27)
pBMI ^1^, kg/m^2^	25.8	5.3	26.5	5.2	23.8	5.5	26.4	5.2	
	**n**	**%**	**n**	**%**	**n**	**%**	**n**	**%**	
pBMI categorization									c^2^ = 11.81 (0.07)
Underweight	5	4.2	0	0.0	1	3.9	4	5.6	
Normal	55	46.6	10	50.0	19	73.1	26	36.1	
Overweight	38	32.2	6	30.0	4	15.4	28	38.9	
Obese	20	17.0	4	20.0	2	7.7	14	19.4	
Education level									c^2^ = 12.79 (0.047) *
High School/GED ^2^	53	44.9	11	55.0	7	26.9	35	48.6	
Vocational/Associates	11	9.3	1	5.0	3	11.5	7	9.7	
Bachelors	31	26.3	5	25.0	5	19.2	21	29.2	
Graduate School	23	19.5	3	15.0	11	42.3	9	12.5	
Race									c^2^ = 26.75 (0.003) **
American Indian/Alaskan	49	41.5	8	40.0	6	23.1	35	48.6	
Asian	3	2.5	0	0.0	3	11.5	0	0.0	
Biracial	4	3.4	1	5.0	2	7.7	1	1.4	
Black/African American	17	14.4	3	15.0	2	7.7	12	16.7	
Other	5	4.2	3	15.0	0	0.0	2	2.8	
White/Caucasian	40	33.9	5	25.0	13	50	22	30.6	
Ethnicity									c^2^ = 14.29 (0.001) **
Hispanic/Latina	79	67.0	19	95.0	11	42.3	49	68.1	
Not Hispanic/Latina	39	33.0	1	5.0	15	57.7	23	31.9	
Household Income in Past Year									c^2^ = 22.88 (0.001) **
$0–$25,000	39	33.1	6	30.0	9	34.6	24	33.3	
$26,000–$50,000	24	20.3	4	20.0	1	3.9	19	26.4	
$51,000–$100,000	29	24.6	9	45.0	3	11.5	17	23.6	
$101,000–≥$250,000	26	22.0	1	5.0	13	50.0	12	16.7	
Prenatal Vitamin Intake									c^2^ = 0.74 (0.69)
Yes	109	92.4	18	90.0	25	96.2	66	91.7	
No	9	7.6	2	10.0	1	3.9	6	8.3	
Number of Children									c^2^ = 3.57 (0.47)
0	48	40.7	7	35.0	9	34.6	32	44.4	
1–2	62	52.5	10	50.0	15	57.7	37	51.4	
≥3	8	6.8	3	15.0	2	7.7	3	4.2	
Relationship with baby’s father									c^2^ = 0.74 (0.69)
Together	109	92.4	18	90.0	25	96.2	66	91.7	
Not together	9	7.6	2	10.0	1	3.9	6	8.3	
Exercise of 30 min or more									c^2^ = 4.38 (0.63)
Rarely/none in past year	25	21.2	6	30.0	5	19.2	14	19.4	
At least 1x month	6	5.1	1	5.0	1	3.9	4	5.6	
At least 1x week	19	16.1	2	10.0	2	7.7	15	20.8	
Two or more × a week	68	57.6	11	55.0	18	69.0	39	54.2	
Current Working Status									c^2^ = 1.96 (0.375)
Yes	65	55.1	9	45.0	17	65.4	39	54.2	
No	53	44.9	11	55.0	9	34.6	33	45.8	

** *p* < 0.01, * *p* < 0.05. ^1^ Pre-pregnancy body mass index. ^2^ General Educational Development Test.

**Table 2:** Average total HEI ^1^ scores.

	Gestational Weight Gain Groups												
	Total Sample n = 118 (100%)			Inadequate n = 20 (17%)			Adequate n = 26 (22%)			Excessive n = 72 (61%)			
	M	SD	Range	M	SD	Range	M	SD	Range	M	SD	Range	*F*_2,115_ (*p*-Value)
Average Total HEI	54.1	10.7	28.8–85.3	55.0	7.2	43.5–72.2	58.4	10.8	43.3–81.5	52.3	11.2	28.8–85.3	3.21 (0.04) *

* *p* < 0.05. ^1^ Healthy Eating Index.

**Table 3:** Final logistic regression of selected predictors on risk of excessive GWG.

Predictors	OR	95% CI		Test Statistic (*p*-Value)
Average HEI ^1^ Score	0.95	0.91	1.01	${\;\chi }_{1}^{2}$ = 2.31 (0.13)
pBMI ^2^	1.05	0.94	1.18	${\;\chi }_{1}^{2}$ = 0.85 (0.36)
Maternal Age	1.03	0.93	1.16	${\;\chi }_{1}^{2}$ = 0.44 (0.51)
Income in past year				$\chi_{3}^{2}$ = 6.34 (0.10)
$26,000–$50,000	10.77	1.02	113.67	
$51,000–$100,000	2.83	0.29	27.81	
$101,000–≥$250,000	0.76	0.06	9.38	
Education				$\chi_{3}^{2}$ = 3.96 (0.27)
Vocational/Associates	0.33	0.05	2.07	
Bachelors	0.92	0.10	8.55	
Graduate School	0.24	0.02	2.90	
Ethnicity	0.84	0.18	3.89	${\;\chi }_{1}^{2}$ = 0.05 (0.82)
Race	1.71	0.43	5.47	${\;\chi }_{1}^{2}$ = 0.44 (0.51)

^1^ Healthy Eating Index. ^2^ Pre-pregnancy body mass index.

**Table 4:** HEI ^1^ component scores.

Gestational Weight Gain Groups													
	Total Sample n = 118 (100%)			Inadequate n = 20 (17%)			Adequate n = 26 (22%)			Excessive n = 72 (61%)			
HEI Components	M	SD	Range	M	SD	Range	M	SD	Range	M	SD	Range	*F*_2,115_ (*p*-Value)
Total Fruits	3.0	1.6	0.0–5.0	3.4	1.6	0.1–5.0	3.4	1.4	0.4–5.0	2.7	1.6	0.0–5.0	2.93 (0.057)
Whole Fruits	2.6	1.8	0.0–5.0	3.1	1.6	0.0–5.0	3.0	1.8	0.0–5.0	2.3	1.8	0.0–5.0	2.26 (0.11)
Total Vegetables	3.2	1.1	0.7–5.0	3.3	0.9	1.5–5.0	3.6	1.2	0.7–5.0	3.0	1.1	1.1–5.0	2.69 (0.07)
Greens & Beans	2.5	1.8	0.0–5.5	2.9	1.7	0.0–5.0	3.5	1.4	0.0–5.0	2.1	1.8	0.0–5.0	6.77 (0.002) **
Whole Grains	2.4	2.2	0.0–10.0	1.8	1.6	0.0–4.9	2.7	2.4	0.0–10.0	2.4	2.3	0.0–10.0	1.02 (0.37)
Dairy	6.1	2.4	0.6–10.0	5.6	2.8	0.6–9.9	5.8	2.5	1.1–10.0	6.3	2.2	1.4–10.0	0.98 (0.38)
Total Protein Foods	4.3	0.8	1.6–5.0	4.4	0.6	3.0–5.0	4.4	0.8	2.8–5.0	4.3	0.8	1.6–5.0	0.06 (0.94)
Seafood & Plant Proteins	2.7	1.6	0.0–5.0	2.6	1.4	0.0–5.0	3.5	1.5	0.0–5.0	2.4	1.6	0.0–5.0	5.37 (0.006) **
Fatty Acids	4.2	2.6	0.0–10.0	4.0	2.4	0.8–10.0	4.7	2.8	0.2–10.0	4.0	2.6	0.0–9.5	0.67 (0.51)
Refined Grains	5.8	2.5	0.0–10.0	6.4	2.0	2.9–10.0	5.7	2.6	0.0–10.0	5.7	2.6	0.0–10.0	0.52 (0.59)
Sodium	3.9	2.3	0.0–9.2	4.2	2.2	0.3–8.2	4.0	2.2	0.0–7.7	3.8	2.3	0.0–9.2	0.26 (0.78)
Added Sugars	7.4	2.1	0.8–10.0	6.8	2.3	2.2–10.0	7.9	1.9	4.1–10.0	7.3	2.1	0.8–10.0	1.63 (0.20)
Saturated Fats	6.1	2.4	0.0–10.0	6.6	1.8	2.7–10.0	6.2	2.6	1.0–10.0	5.9	2.5	0.0–10.0	0.84 (0.44)

** *p* < 0.01. ^1^ Healthy Eating Index.

**Table 5:** Multiple logistic regression of the HEI ^1^ components on risk of excessive GWG ^2^.

HEI Component Scores	OR	95% CI		Test Statistic (*p*-Value)
Total Fruits	0.81	0.57	1.15	${\;\chi }_{1}^{2}$ = 1.42 (0.24)
Whole Fruits	0.93	0.69	1.24	$\chi_{1}^{2}$ = 0.27 (0.60)
Total Vegetables	0.74	0.48	1.14	$\chi_{1}^{2}$ = 1.80 (0.18)
Greens & Beans	0.61	0.43	0.86	$\chi_{1}^{2}$ = 8.07 (0.004) **
Whole Grains	1.05	0.84	1.30	${\;\chi }_{1}^{2}$ = 0.18 (0.67)
Dairy	1.15	0.93	1.43	$\chi_{1}^{2}$ = 1.59 (0.21)
Total Protein Foods	0.73	0.38	1.41	$\chi_{1}^{2}$ = 0.86 (0.35)
Seafood & Plant Proteins	0.60	0.42	0.86	$\chi_{1}^{2}$ = 7.84 (0.005) **
Fatty Acids	0.95	0.78	1.14	$\chi_{1}^{2}$ = 0.32 (0.57)
Refined Grains	0.99	0.81	1.20	$\chi_{1}^{2}$ = 0.02 (0.89)
Sodium	0.97	0.79	1.19	$\chi_{1}^{2}$ = 0.07 (0.78)
Added Sugars	0.86	0.66	1.12	$\chi_{1}^{2}$ = 1.21 (0.27)
Saturated Fats	0.96	0.79	1.16	$\chi_{1}^{2}$ = 0.18 (0.67)

** *p* < 0.01. ^1^ Healthy Eating Index, ^2^ gestational weight gain.

## Data Availability

The original data presented in this study are openly available in Columbia University Libraries’ Academic Commons at https://doi.org/10.7916/bcr3-f482.

## Abbreviations

The following abbreviations are used in this manuscript:

GED	General Educational Development Test
GWG	Gestational weight gain
HEI	Healthy Eating Index
IOM	Institute of Medicine
NRC	National Research Council
pBMI	Pre-pregnancy body mass index
CUIMC	Columbia University Irving Medical Center
ASA24	Automated self-administered 24 h dietary recall
NCI	National Cancer Institute
WIC	Special Supplemental Nutrition Program for Women, Infants, and Children
